# Smallpox

**DOI:** 10.1093/infdis/jiaa588

**Published:** 2021-09-30

**Authors:** Joel G Breman

**Affiliations:** Fogarty International Center, National Institutes of Health, Bethesda, Maryland, USA

You have erased from the calendar of human afflictions one of its greatest. Yours is the comfortable reflection that mankind can never forget that you have lived. Future nations will know by history only that the loathsome smallpox has existed and by you has been extirpated.

Thomas Jefferson, writing to Edward Jenner in 1806 [1].

Before knowledge and use of vaccines, protection against smallpox was practiced more than a thousand years ago by traditional approaches. Invoking the good graces of smallpox gods, goddesses, and saints by individuals and communities was common [2]. Isolation of patients was the only means known to appease and contain the bad spirits that brought and spread the disease.

Traditional medical practitioners in some areas of China, India, Egypt, Ethiopia, and elsewhere collected materials from the pustules or crusts of the afflicted and inserted these into the noses or skin of healthy persons seeking protection [2]. This procedure, called inoculation or variolation, probably had little effect on curtailing epidemics because of its limited use and variability of potency of the inoculum.

It is remarkable that some early inoculators inserted scabs into the nose, without understanding that smallpox is acquired via the respiratory route, and, that scratching pustular material into the skin could have the same salutary effect. Nasal inoculation or dermal variolation, using material containing live virus, resulted sometimes in mild illness and protection. However, some cases of smallpox in recipients had the potential to spread within persons and communities.

## EARLY VACCINES

Edward Jenner, the country doctor from Berkeley, Gloucester, England, is recognized as the father of smallpox vaccination. Jenner’s 1796 observations, that cowpox protected against smallpox when scratched into the skin of recipients, were written up in detail and presented to the Royal Society of England in 1798 and promoted widely in letters [3]. Yet Jenner was not the first to make these observations.

Some historians note that John Fewster and others, as early as 1768, living near Jenner in Thornbury, and Benjamin Jesty in 1774 in Westminster, United Kingdom, observed the benefits of cowpox inoculation for protecting humans against smallpox [4]; these observations were not documented and disseminated, however, so they remain in obscurity.

Jenner promoted vaccines in England and elsewhere by letters and speeches and by giving vaccinations gratis to local residents at the “vaccine hut” outside his home (The Chantry). There was immediate fierce opposition by persons who believed that biological products from cows would result in growths resembling cows on the bodies of recipients.

People of influence had an early role in supporting both inoculation and vaccination. Lady Mary Wortley Montagu, wife of the UK Ambassador to Turkey, who had had smallpox in England, observed variolators in Turkey performing inoculations. She was so impressed that she promoted the procedure via a series of letters starting in 1717 [1, 2]. Thomas Jefferson, Benjamin Franklin, and Benjamin Waterhouse, the latter of Harvard University, were early advocates of vaccination in the United States.

For the next 100 years, technical problems tied to vaccine quality impeded the successful use of vaccine globally. Well into the 20th century, the major challenges were growing adequate quantities of vaccine of measurable potency, sterility, and durability despite differences in temperature, climate, and humidity [5].

Mode of administration was another challenge. Different scratch and inoculation techniques were used, particularly in India [6]. Throughout much of the 1800s vaccine was passed from arm to arm or dried and put on small “points” (sharp objects of ivory, steel). These methods were not reliable. Toward the end of the 19th century, animals, especially the skin of living cows, were used to grow the virus used for vaccination [5].

During the 19th century, arm-to-arm vaccination was the standard method of maintaining the product’s efficacy, even during long voyages. Some practitioners put threads through the pustular material. The threads were dried and sent to the areas for populations to be vaccinated; potency certainly waned during such travel. On long sea voyages, groups of orphan children were often sent specifically to assure arm-to-arm transfer of the pustular material. In the early 1900s, an attempt to dry and preserve vaccine for shipment from France to their colonies in West Africa was described by Fasquelle and Fasquelle [7].

## VACCINATION AFTER 1900

Pustular material from cows or patients with pustular disease of indeterminate origin was used for more than a century as the source of smallpox vaccine. By the beginning of the 1900s, vaccination against smallpox was being practiced in most industrialized countries. The virus now used, called vaccinia, has an obscure origin. The product may have originally been a hybrid between cowpox virus and variola virus or some other orthopoxvirus by serial passage in artificial conditions, or, as Baxby posits, vaccinia may be a laboratory survivor of a virus now extinct [8], p 214]. The various vaccinia strains globally are similar to each other genetically but differentiated from other poxviruses, including cowpox and variola viruses, by DNA mapping. By the 1950s there was improvement in vaccine quality, distribution, and public health infrastructure. Smallpox was virtually eliminated from Europe and North America by that time.

Since Jenner’s time opponents of vaccination have based their concerns on perceived physical harm from the procedure and breaching of individual rights. Over time, vaccination has been considered a public health good and inserted into law in the United States and elsewhere and upheld by the Supreme Court [9].

## ERADICATION STRATEGY EVOLUTION

High vaccination coverage had been the strategy of national and international smallpox control and elimination strategies since Jenner’s findings slowly spread worldwide and became accepted in the 1800s. However, the continued existence of the disease on virtually all continents was due to fragmented and inadequate health systems. Access to remote populations was impossible in many areas and acceptance of evolving vaccine production and delivery technology was slow. Most importantly, the colonial legacy starting in the late 1800s left many areas of the world dependent on European control and resources for their health and other programs, particularly in Africa. Conservation of liquid vaccine produced mainly on cows was very difficult, because refrigeration was virtually nonexistent in the tropics until the mid- to late-1900s.

In West and Central Africa and India more vaccinations were given than the censused population, yet smallpox raged because of poor vaccine quality. Massive epidemics of smallpox appeared periodically in virtually all tropical countries and several areas of temperate countries well into the 1900s, fueled by high levels of susceptibility as a result of new births, those who received poor quality vaccine, and nonimmune older persons [10].

The World Health Organization (WHO) was formed in 1948 with a mandate to develop public health policies and to coordinate surveillance, and some control and eradication initiatives. By the 1950s, many countries had passed public health laws and implemented smallpox vaccination programs, many of which were successful, particularly in the northern hemisphere. In 1959, the representative of the Soviet Union proposed a resolution for a global smallpox eradication program to the World Health Assembly [2]; this was based, in part, on the outbreaks of smallpox in several of the southern republics, which underscored the priority for development of a potent vaccine to control the outbreaks. In addition, the Soviets wished to provide vaccines to the WHO as a gift to the global program. Yet, little progress was made over the next 6 years toward global eradication [11], p 334].

Between 1959 and 1966, few funds were received or invested by WHO for smallpox eradication, and few staff were assigned to the program. The strategy was entirely reliant on attempting to achieve 80% vaccination coverage. Each country had to rely on its own manufacturers or products acquired via the WHO, mainly from Soviet donations. The Soviet product caused severe adverse reactions, which probably was responsible for poor acceptance and coverage, especially in India, where tens of millions of doses were sent [2].

Despite the 1959 resolution, WHO internal and external support for the program languished until the middle 1960s. In 1966, the United States, backed by President Lyndon Johnson, supported another resolution in support of an intensified smallpox eradication program [11], p 334].

## ERADICATION AND VACCINE QUALITY

By the late 1960s, when the intensified global smallpox eradication program began, it was found by WHO, that vaccine was being produced by many different countries using varying procedures. A major step was taken initially by the Smallpox Eradication Unit at WHO to advise standardized production methods and international quality control of vaccines used in the global program [2].

One of the first steps of the intensified program was to do a detailed survey of vaccine production procedures, quality, and production capacity in endemic and nonendemic countries. Questions about the relatively newly perfected freeze-dried vaccine methods, strains used, methods of growing virus, and bottling (doses per vial) were assessed [2, 12].

Of 72 laboratories assessed, 59 replied to the WHO survey. Fifty-one laboratories (86.4%) harvested vaccinia virus from the skin of calves or sheep, and 6 (10.1%) from water buffaloes; 3 also reported using chick embryos, and 3 used tissue culture. Of the 59 laboratories, 23 (39.0%) used Lister strain (origin UK), 6 (10.2%) New York City Board of Health strain, 7 (11.9%) Paris strain, and 22 (37.3%) a variety of strains; one reported using a mixture of vaccinia and cowpox.

## VACCINE POTENCY, STABILITY, AND BACTERIAL CONTENT IN 1967

When the WHO requested laboratories producing smallpox vaccines to submit information on potency, heat stability and bacterial content, the following was received from 59 laboratories from 4 WHO regions. Of the 59, 31 (52.5%) reported that all 3 recent product lots were satisfactory (titers of ≥10^8.0^ pock-forming units on chicken chorioallantoic membranes), and 16 (27.1%) reported vaccine stability after 4 weeks at 37°C; 12 of 53 laboratories (22.6%) sending data reported bacterial counts >500/mL, which were unacceptable by WHO standards.

After the establishment of the independent WHO reference centers for smallpox vaccine testing at Connaught Laboratories in Toronto and the National Institute of Public Health in Bilthoven, the Netherlands, it was concluded that, of 39 batches submitted by producers intending to develop freeze-dried vaccine for use in their own countries and in the global programs, 25 (64.1%) failed to meet standards. The conclusion was that, in 1967, not more than 10% of the vaccine in use in endemic countries met WHO requirements. Freeze-dried vaccine, a procedure credited to Leslie Collier, rendered the vaccine stable for long periods and could be reconstituted with diluent in the field [12].

The WHO smallpox unit established (1) a manual on the production of freeze-dried vaccine, (2) a traveling set of experts who gave seminars on vaccine production in laboratories, (3) training that including hands-on demonstration of the production of reference smallpox vaccines, (4) provision of seed lots of Lister strain vaccinia, (5) development of a heat stability test, and (6) guidance for regular testing of vaccine potency and heat stability to be used by the reference centers.

## ADMINISTRATION OF VACCINE

Another reason earlier smallpox campaigns failed was the inadequacy of the instruments used to immunize. Baxby has reviewed the variety of instruments, many of which resembled “tools of torture” [13]. Some of these required large amounts of liquid vaccine and caused maceration of the skin. This often resulted in infections in the tissues of recipients, poor success rates, and refusal of the procedure.

**Figure 1.**
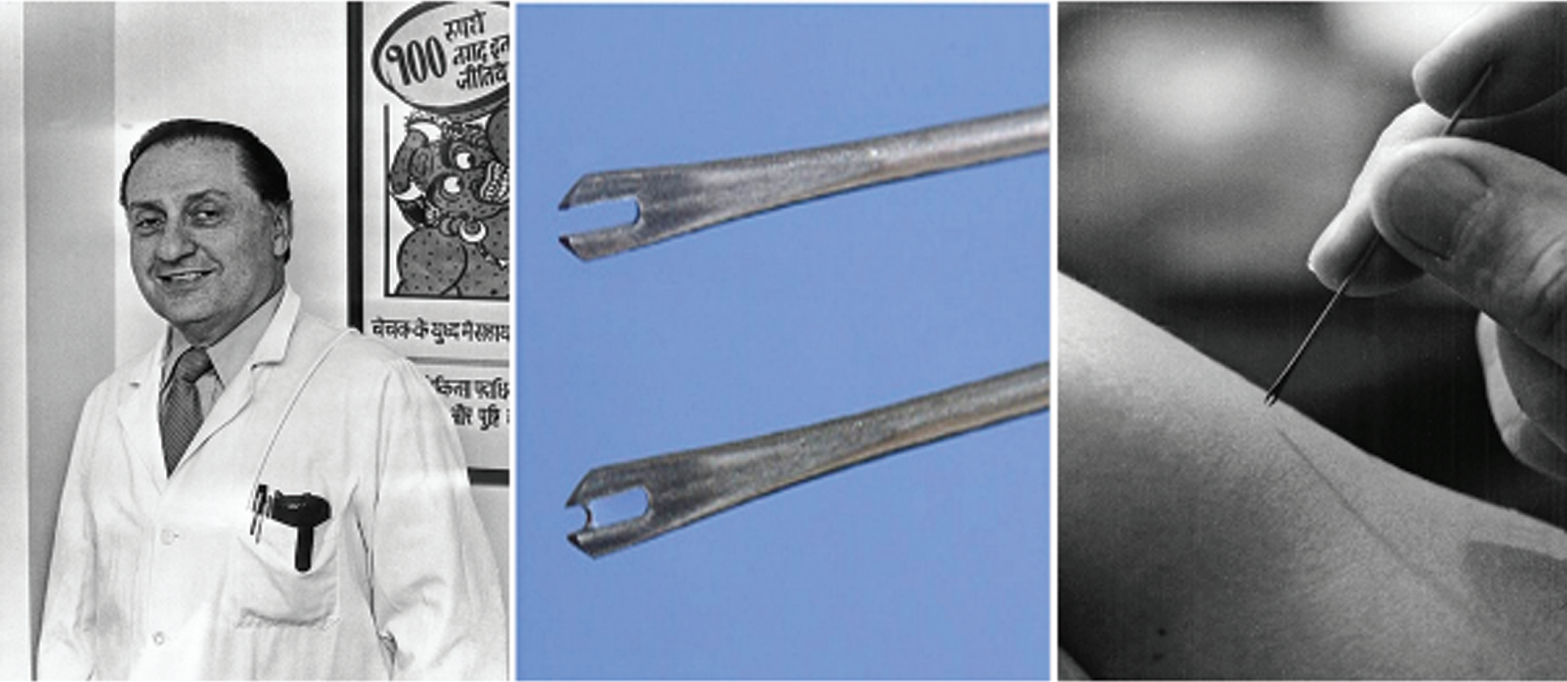
Bifurcated needle for intradermal injection of vaccinia virus, invented by Benjamin Rubin (left) of Wyeth Laboratories. (Source: Fenner et al [2], WHO.)

**Figure 2.**
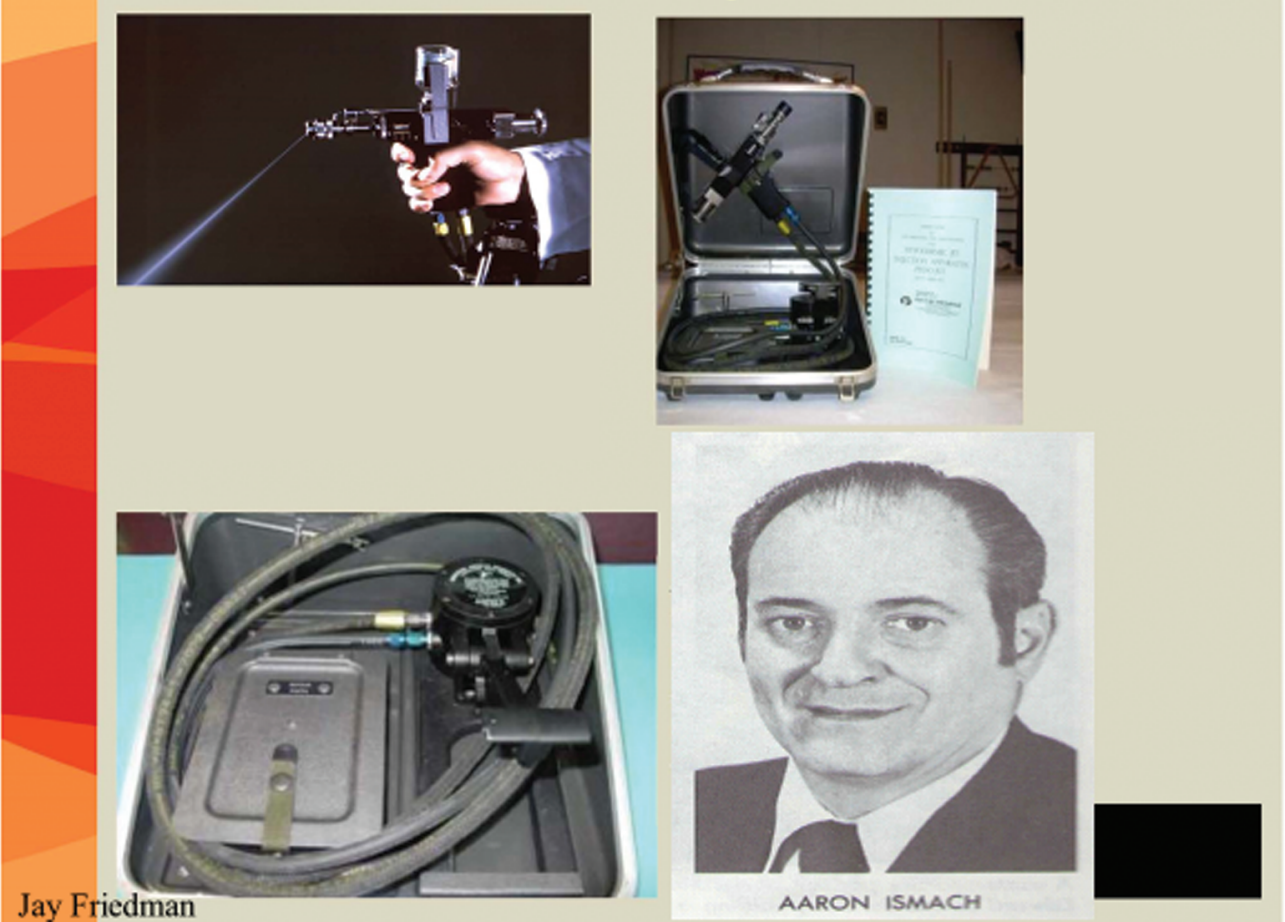
Jet injector for intradermal injection of vaccinia virus. *Top left,* Intradermal nozzle squirts at an angle for smallpox vaccination. *Top right,* Jet Injector in its case. The instruction book for West Africa was in English and French. *Bottom right,* Aaron Ismach, who is credited with the design of this injector. (Source: WHO, CDC)

The 2 most effective tools for injecting vaccinia virus intradermally during the eradication program were the bifurcated needle and the jet injector gun (Figures 1 and 2). The bifurcated needle is a 2-pronged adaptation of a sewing needle, invented by Benjamin Rubin of Wyeth Laboratories. The sterile needle was dipped into a reconstituted vial of vaccine; a drop of vaccine was caught between the prongs. The needle is jabbed rapidly 15 times into the upper deltoid region of the arm until a small drop of blood or serum appears. The jabs should all be within a 1-cm-diameter area. Acetone is preferable to alcohol for cleansing the arm because it dries quickly; alcohol could inactivate the vaccinia virus if not dry when the multiple punctures are made. The needles are kept in a durable tubular container with a hole to shake out a sterile metal needle when needed. The needles are cleaned after use, placed in a tub of boiling water for 20 minutes, cooled, and replaced in the plastic containers, ready for reuse.

**Figure 3.**
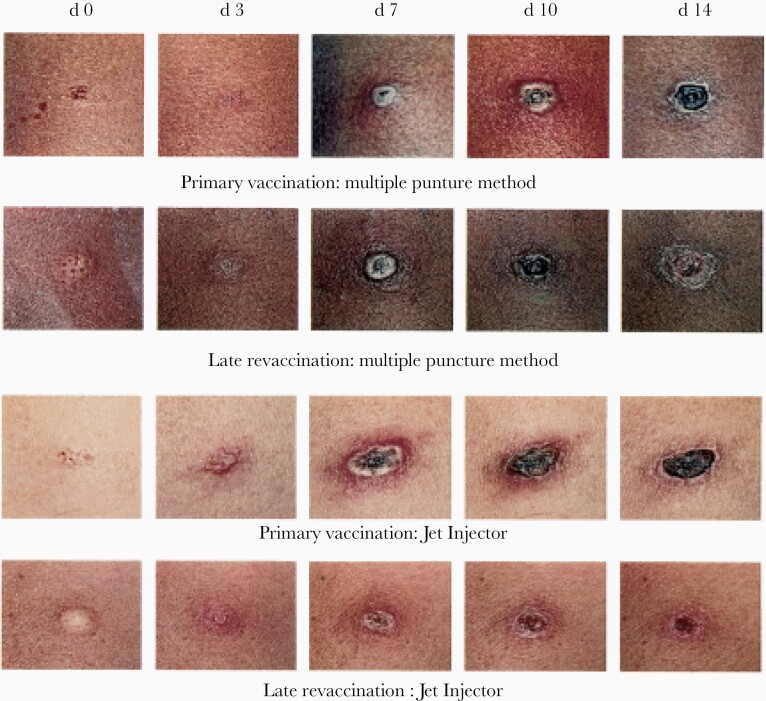
Skin reaction after primary vaccination and late revaccination (several years later), performed using a multiple puncture method or a jet injector. (Source: CDC.)

The jet injector is a pneumatic foot-activated apparatus (gun) that injects smallpox vaccine intradermally via a special nozzle. It was most effective in places where large groups of people could be assembled, such as Brazil and West and Central Africa. Aaron Ismach of the US Army is credited with the design. The apparatus has been criticized for requiring frequent maintenance and spare parts; however, in Guinea, for example, 6 vaccination teams averaged close to 2000 vaccinations per working day over a 2-year period in a rural environment where people could be assembled; meticulous attention was given to daily maintenance [14]. Not only was coverage high with both of these devices during the smallpox eradication campaign, but the success (“take”) rates after vaccination were >98% in primary vaccines and 95% in those receiving revaccination (see Figure 3).

Smallpox vaccine multiplies in the skin’s epithelium, producing a slight fever and characteristic skin reaction with redness and induration leading to a pustule by about day 7 in those receiving primary vaccines; this “Jennerian pustule” usually starts to crust and desquamate by day 14, leaving a scar. Induration may occur after revaccination; if many years have passed since vaccination, a typical Jennerian vesicle will occur (Figure 3).

## VACCINE COMPLICATIONS

**Figure 4.**
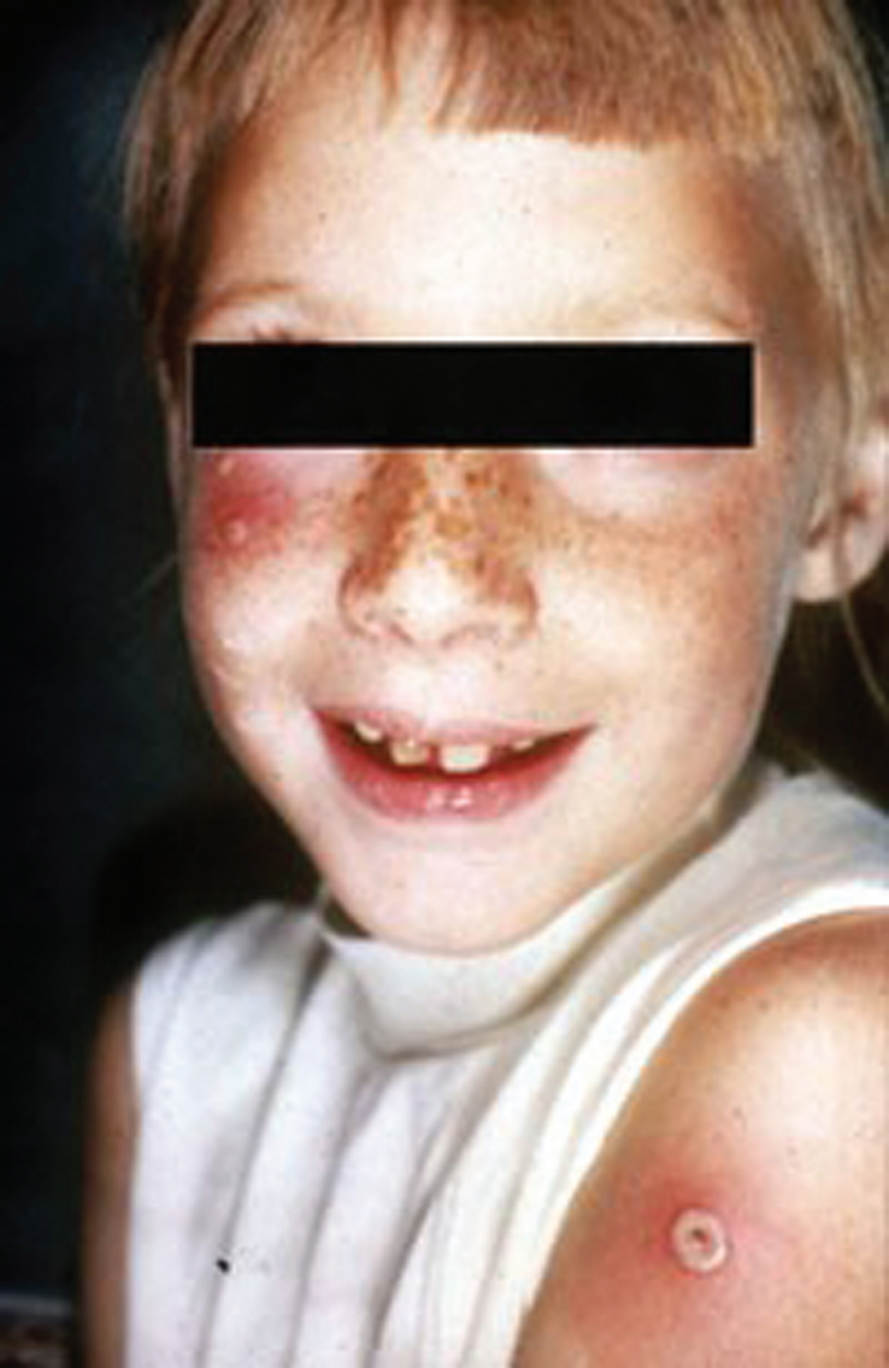
Accidental autoinoculation of cheek with vaccinia virus, approximately 5 days old. Primary take with “Jennerian vesicle” on arm, 10–12 days old. (Photograph courtesy of John M. Leedom, MD, CDC collection.)

During the intensified eradication program, a major effort was made to assess take rates. Only in the United States was a comprehensive nationwide survey done to look at adverse events following smallpox vaccination with the NY Board of Health seed strain. In 1970 and 1971, Lane et al published articles on complications, dividing United States recipients into those receiving smallpox vaccine for the first time (primary vaccinees) and those receiving the vaccine as “revaccinees” [15, 16, 17]. Data from the 10-state survey were collected by more active ascertainment of adverse events, resulting in 5 times the number of complications reported by the passively reported events [15]. The most severe conditions were postvaccinal encephalitis and generalized vaccinia (Figure 4). The death rate was about 1 in 1 million vaccinations in those receiving primary vaccines, mainly young children. Children with immunoglobulin deficiencies or severe eczema were prone to adverse events. Screening before vaccination could have decreased the number of complications.

In 1971, the US Advisory Committee on Immunization Practices recommended that routine smallpox vaccination be stopped in the United States. This was based on the assessment of the risks of those being vaccinated and the extremely low chance of importation, even though smallpox remained endemic in East Africa and the Indian subcontinent [18, 19].

Following the certification of smallpox eradication by the World Health Assembly in 1980, the WHO advised all countries to stop routine smallpox vaccination [2]. This was accepted by virtually all countries in the early 1980s. In 2002–2003, after the terrorist airplane attacks on the Twin Towers buildings in New York City and the anthrax mailings and deaths due to a domestic bioterror event, a limited number of smallpox vaccinations were given in the United States, mainly to first responders. The US military was vaccinated, and acute myopericarditis and cardiac arrhythmias developed in 37 of >400 000 recruits [17, 18].

## SMALLPOX: THE DISEASE

There were 2 major manifestations of smallpox: *Variola major* and *Variola minor. V. major* was seen mainly in the Indian subcontinent, and parts of Africa and Asia during the eradication program and was the most severe form, with a 30% fatality rate. *V. minor,* observed in east of Africa and Latin America, was milder, with fewer lesions and a case fatality rate of <5%. The clinical and epidemiological features of smallpox have been well covered in recent and past literature and are summarized in Tables 1 and 2 [2, 20]. The eruption evolves with all skin lesions at the same stages at a given point of time, starting from macules and papules, followed by pustules, vesicles, and finally crusts, over a 10–20-day period. 

**Table 1.** Clinical and Epidemiological Features of Smallpox and Considerations for Certification

Feature	Indications of Smallpox
Incubation period	14–17 d
Syndrome visible	Yes (eruption)
Recognized by public	Yes (eruption)
Asymptomatic carriers	None
Transmission mode	Respiratory
Vector/reservoir	Human
Secondary attack rate among susceptible persons	High (40%–90%)

**Table 2.** Characteristics of Surveys to Certify Eradication of Smallpox

Characteristic	Comment
Clinical features surveyed	Fever and rash; facial pockmarks
Target group	School-age children
Diagnostic alternatives	Chickenpox, monkeypox
Environmental surveys for microbe	No
Laboratory confirmation	Yes
Rumor registers	Yes
Reward for reporting cases	Yes
Minimum interval since last case before certification	2 y
Continuing research	Yes

**Figure 5.**
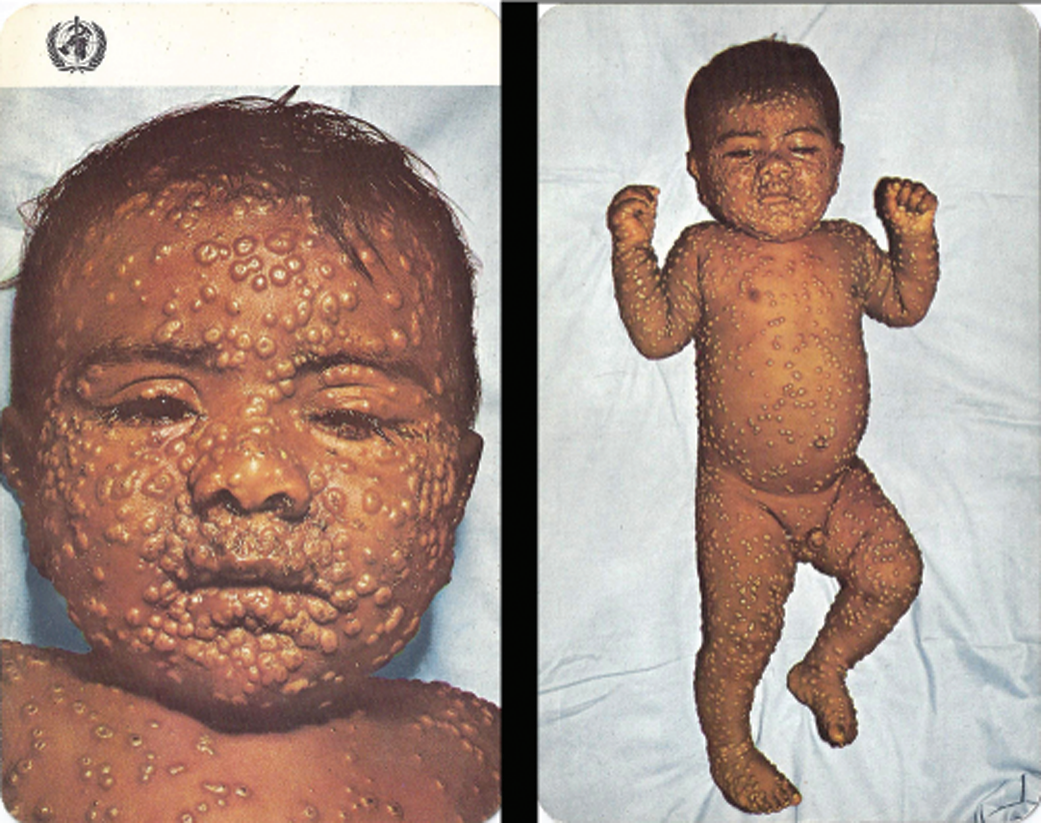
Smallpox on day 8 of eruption (World Health Organization photograph).

The lesions of smallpox are focused on the peripheral (centrifugal) parts of the body (Figure 5), in contrast to the rashes of diseases like chickenpox which have centripetal distribution. The most common diagnostic dilemma is in the differential diagnosis is chickenpox which can be severe in older persons and immunocompromised patients [20]. Today, in Africa the eruption of human monkeypox cannot be easily distinguished from smallpox except by laboratory testing; patients with monkeypox often have cervical and inguinal lymphadenopathy [21].

## THE ERADICATION PROGRAM

The initial strategy of the intensified smallpox eradication program beginning in 1967 was based on at least 80% vaccination coverage of the population in each country. This strategy, while successful in northern countries where vaccine quality was monitored and people were told to be revaccinated every 3 years, was not effective in the heavily endemic areas. These countries were mainly in Africa, Latin America, and Asia. In Africa, the health infrastructure was poor, because many countries became newly independent in the late 1950s and 1960s and had limited resources. Some countries had a tradition of mobile health teams trained to detect perils, such as sleeping sickness, leprosy, and onchocerciasis, and to give smallpox vaccines. The countries did not often have managerial expertise or refrigerated repositories to conserve vaccines and other heat-sensitive biologicals [22, 23].

In the mid-1960s President Lyndon Johnson committed the United States to supporting an 18-country West and Central Africa smallpox eradication-measles control program. This followed a visit to the United States by the minister of health of Burkina Faso (then Upper Volta), who learned about the newly developed measles vaccine and wanted Africa to benefit. The funding came via the US Agency for International Development. The US Centers for Disease Control and Prevention (CDC; then the Communicable Disease Center) was the implementing organization.

Of the 11 countries with the highest incidence of smallpox, 7 were in West and Central Africa. Two major innovations in this program have changed the face of public health. The first was the use of operational specialists from the CDC—managers responsible for organization, finances, equipment, supplies, logistics, transportation—who were assigned to countries with medical epidemiologists, both of whom worked closely with national counterparts [24], p 141].

Second, the concept of surveillance containment was rediscovered and refined in Nigeria [25]. Intensified surveillance and ring vaccination became the major strategies used throughout the program, especially in the Indian subcontinent [2, 26, 27].

## SURVEILLANCE CONTAINMENT AND RING VACCINATION

Using epidemiological information, rumor notices, and village-by-village searches, persons with suspected smallpox were identified and confirmed virologically when outbreaks were few. Patients were isolated in or near their home residence; food was supplied, and a 24-hour guard hired by the program to assure the patient did not circulate until the crusts had fallen. All primary contacts of the patient since the illness began were identified and vaccinated, as were residents of neighboring houses and villages within 5 km. 

Detailed maps and censuses of houses and residents in the villages within 5 km of the patient were made and used to assure all occupants had been vaccinated. Cross-notification was done, by telegram or phone, to health authorities both from places where case patients had probably acquired their disease and from places they had visited since their infections first manifested. Secondary areas of priority (nearby villages, markets, schools, assembly areas) were identified on hand-drawn maps and were visited, and residents were interrogated and vaccinated. This strategy is also called “ring vaccination,” as concentric circles or areas of priority were often mapped by program authorities.

In the Indian subcontinent, the active case search approach was highly refined. At one time 150 000 field workers were going from village to village [26, 27]. Major increases in numbers of infected villages and cases were found, compared with routine reporting. The largest exportation of cases in the program occurred in India. The Tatanagar railway station was the source of dozens of cases in surrounding states and districts until the case tracking and containment strategy was intensified, with major assistance from Tata industries that joined the program with staff and funds [27]. In Bangladesh, it was found by facial pockmark surveys that <5% of the actual cases were being reported before the program began [28].

**Figure 6.**
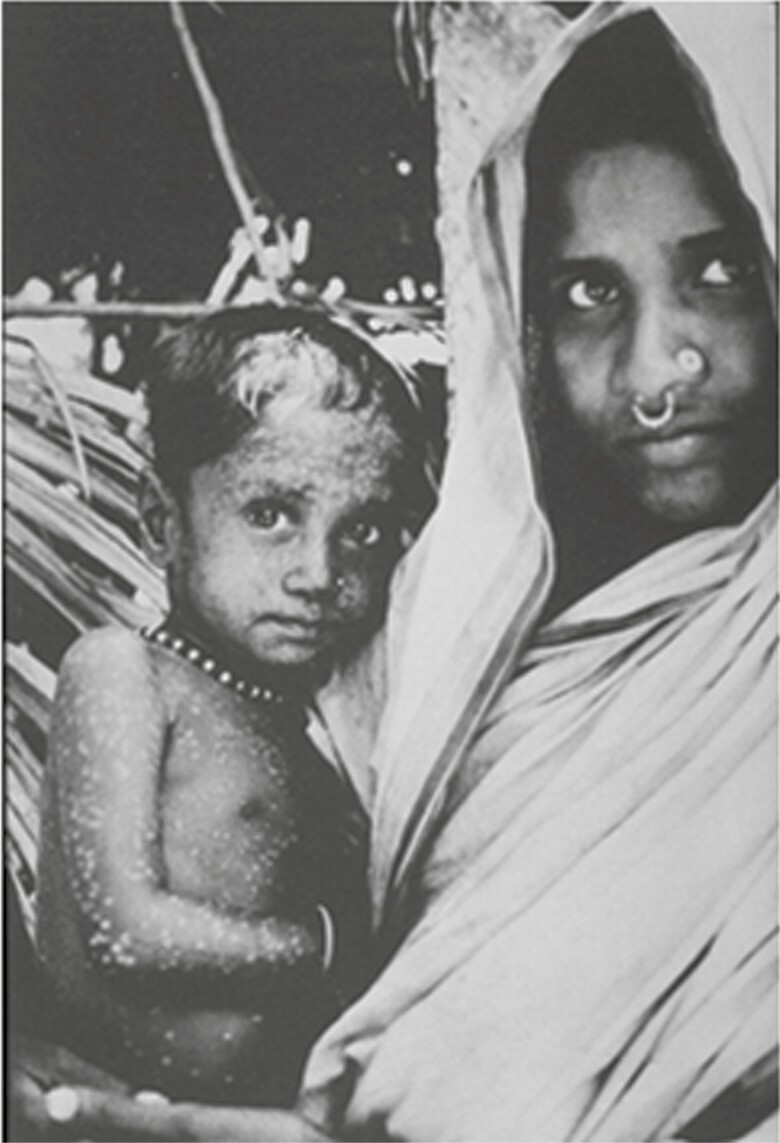
Last case patient with *Variola major* smallpox (Bangladesh, 1975). (Source: World Health Organization.)

**Figure 7.**
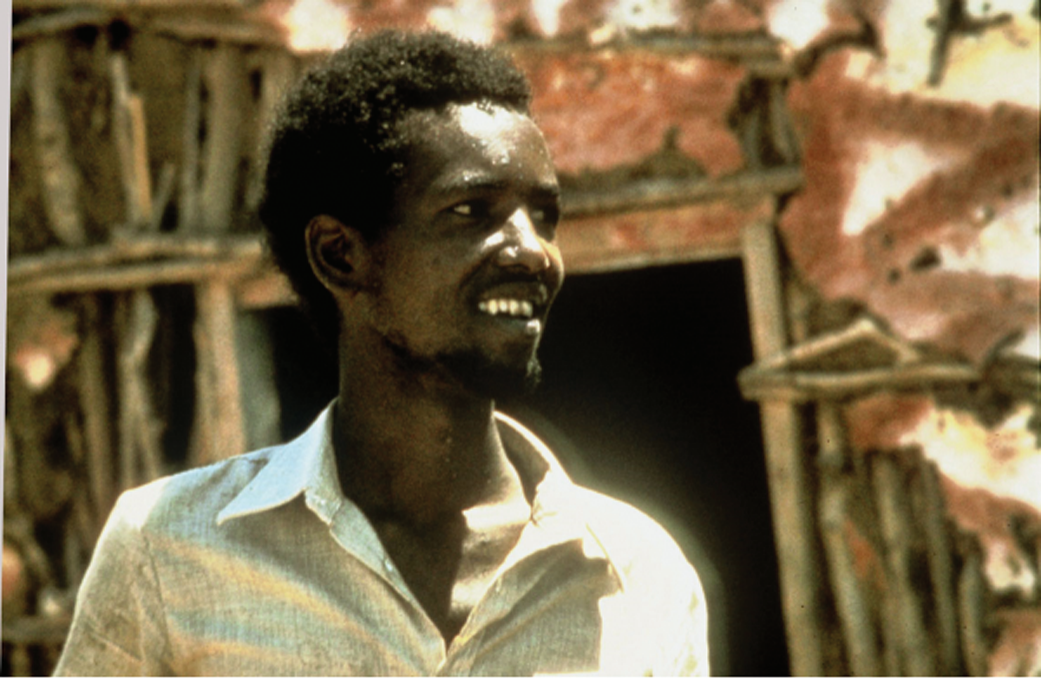
Last case patient with naturally transmitted smallpox (October 1977). (Source: World Health Organization.)

The last case caused by naturally occurring transmission of *Variola major* occurred in Bangladesh in October 1975, and the last of disease *Variola minor* in Somalia on 26 October 1977 (Figures 6 and 7). In 1978, an outbreak of smallpox occurred in Birmingham, United Kingdom, associated with a laboratory working with poxvirus variola virus: the ducting system connecting the laboratory to a photographer’s office above was the conduit of contamination [2].

## CERTIFICATION

After a series of independent evaluations, including field visits to formerly endemic counties by separate international commissions, the Global Commission for the Certification of Smallpox Eradication concluded that eradication had been achieved in December 1979 (Tables 3 and 4). The number of laboratories with variola virus was reduced from 76 to 6 by May 1980, when the World Health Assembly accepted the recommendations of the Global Commission that eradication had indeed been achieved [29]; there are now only 2 laboratories known to retain variola virus stocks—Vektor in Novosibirsk, Russia, and at the CDC in Atlanta. Both laboratories are visited every 2 years by biosafety experts convened by WHO, to assure that maximum biocontainment of the variola isolates is assured.

**Table 3.** Smallpox Certification Activities in 200 Countries, 1977–1980^a^

Countries by Category	Countries, No.	Population (Billions)
Total	200	4.5
Submitting statements	121	1.5
Visited by commissions	79	3.0
At special risk	44	1.8
Endemic during smallpox eradication programs	35	1.2

^a^A total of 17 000 specimens were collected.

**Table 4.** Smallpox Certification Activities in India: Facial Pockmark Surveys, 1977

Age Group	Survey	Persons Examined, No.	
		Total	With Facial Pockmarks^a^
Preschool children	National surveys	271 897	0
	International Commission	3139	0
School-age children	National surveys	224 297	38
	International Commission	26 665	18
Adults	National surveys	1 451 125	650
	International Commission	14 307	94

^a^Pockmarks due to smallpox contracted before 1975.

## CONTINUING ISSUES

There remains concern that a bioterrorist event using smallpox virus could occur; this would wreak havoc globally [30]. The CDC has a smallpox response plan that focuses on national leadership, community-based planning, public health response actions, and health care facility response activities. The US national vaccine stockpile maintains 3 vaccines [31, 32, 33]. ACAM2000 is a Food and Drug Administration–licensed vaccine grown on tissue culture, derived from the New York Board of Health strain of vaccine used to make Dryvax; this latter product was used widely in the eradication program. Aventis Pasteur smallpox vaccine is a vaccine supply created in the 20th century but still retaining potency. The Imvamune vaccine (Bavarian Nordic) uses modified vaccinia Ankara, a nonreplicating vaccine that requires 2 injections and is thought to elicit fewer adverse events, especially in immunologically deficient persons; it is not yet licensed. 

The Food and Drug Administration has recently approved Tecovirimat for the treatment of smallpox; this drug is also destined for the US national stockpile [34]. The WHO, likewise, has a smallpox preparedness plan. Their operational framework addresses education, laboratory diagnosis, biosafety and security, provision of expertise and supplies, and the strengthening of national level responses. WHO also maintains an emergency vaccine stockpile.

The de novo synthesis of horsepox virus has raised concern that a terrorist could recreate variola virus [35]. Because routine smallpox vaccination stopped in the United States in 1971 and globally in the early 1980s, there is justifiable concern over population vulnerability. Constant vigilance for dealing with a return of smallpox is warranted. The 2 high security repositories of variola virus stocks, at CDC in Atlanta, and at Vektor in Novosibirsk, Russia, are inspected periodically by WHO. Periodic debates at the World Health Assembly over whether to destroy the remaining known stocks of smallpox virus have not concluded it was time to do so.

Another important continuing issue is the increasing number of outbreaks and cases of human monkeypox in central and western Africa [36, 37]. These outbreaks will increase as population immunity falls. The smallpox vaccine protects against monkeypox. At some point, consideration must be given whether vaccinia should be used for vulnerable populations in monkeypox-endemic areas.

## Supplementary Data

Supplementary materials are available at *The Journal of Infectious Diseases* online. Consisting of data provided by the authors to benefit the reader, the posted materials are not copyedited and are the sole responsibility of the authors, so questions or comments should be addressed to the corresponding author.

The complete references are available as online Supplemental Material.

## Supplementary Material

jiaa588_suppl_Supplementary-Material

## Notes

***Acknowledgments.*** Thanks to Leigh Henderson, PhD, for help with graphics and Mantra Singh for secretarial assistance.

***Supplement sponsorship.*** This supplement is sponsored by the Bill and Melinda Gates Foundation.

***Potential conflicts of interest.*** Author certifies no potential conflicts of interest. The author has submitted the ICMJE Form for Disclosure of Potential Conflicts of Interest. Conflicts that the editors consider relevant to the content of the manuscript have been disclosed.
