# Supplementary material for: Smallpox
Source: J Infect Dis. 2021 Sep 30;224(Suppl 4):S379–86. doi: 10.1093/infdis/jiaa588 (PMC8482028; doi:10.1093/infdis/jiaa588)
Supplement: jiaa588_suppl_Supplementary-Material [file jiaa588_suppl_supplementary-material.docx]

1. Hopkins DR. The greatest killer: smallpox in history. Chicago, IL: University of Chicago Press, **2002**.

2. Fenner F, Henderson DA, Arita I, Ježek Z, Ladnyi ID. Smallpox and its eradication. Geneva, Switzerland: World Health Organization, **1988**.

3. Jenner E. An inquiry into the causes and effects of the variolæ vaccinæ, a disease discovered in some of the western counties of England, particularly Gloucestershire, and known by the name of the cow pox. London, England: Sampson Low, **1798**.

4. Boylston AW. The myth of the milkmaid. N Engl J Med **2018**; 378:414–5.

5. Esparza J, Lederman S, Nitsche A, Damaso CR. Early smallpox vaccine manufacturing in the United States: introduction of the “animal vaccine” in 1870, establishment of “vaccine farms”, and the beginnings of the vaccine industry. Vaccine **2020**; 38:4773–9.

6. Baxby D. Two hundred years of vaccination. Curr Biol **1996**; 6:769–72.

7. Fasquelle R, Fasquelle A. A propos de l’histoire de la lute contre la variola dan les pays d’Afrique francophone. Bull Soc Pathol Exot **1971**; 64:734–56.

8. Baxby D. Jenner’s smallpox vaccine: the riddle of vaccinia virus and its origin. Vol 14. London, United Kingdom: Heinemann Educational Books, **1981**.

9. Albert MR, Ostheimer KG, Breman JG. The last smallpox epidemic in Boston and the vaccination controversy, 1901–1903. N Engl J Med **2001**; 344:375–9.

10. Breman JG, Alecaut AB, Lane JM. Smallpox in the Republic of Guinea West Africa. I. History and epidemiology. Am J Trop Med Hyg **1977**; 26: 756–64.

11. Henderson DA. Smallpox—the death of a disease: the inside story of eradicating a worldwide killer. Amherst, New York: Prometheus Books, **2009**.

12. Collier LH. Appropriate technology in the development of freeze-dried smallpox vaccine. WHO Chron **1980**; 34:178–9.

13. Baxby D. Smallpox vaccination techniques. 2. Accessories and aftercare. Vaccine **2003**; 21:1382–90.

14. Breman JG, Alecaut AB, Malberg DR, Charter RS, Lane JM. Smallpox in the Republic of Guinea, West Africa: II. Eradication using mobile teams. Am J Trop Med Hyg **1977**; 26:765–74.10.

15. Lane JM, Ruben FL, Neff JM, Millar JD. Complications of smallpox vaccination, 1968: results of ten statewide surveys. J Infect Dis **1970**; 122:303–9.

16. Lane JM, Ruben FL, Neff JM, Millar JD. Complications of smallpox vaccination, 1968: national surveillance in the United States. N Engl J Med **1969**; 281:201–8.

17. Lane JM, Millar JD, Neff JM. Smallpox and smallpox vaccination policy. Annu Rev Med **1971**; 22:251–72.

18. Grabenstein JD, Winkenwerder W Jr. US military smallpox vaccination program experience. JAMA **2003**; 289:3278–82.

19. McNeil MM, Duderstadt SK, Sabatier JF, Ma GG, Duffy J. Vaccination and risk of lone atrial fibrillation in the active component United States military. Hum Vaccin Immunother **2019**; 15:669–76.

20. Breman JG, Henderson DA. Diagnosis and management of smallpox. N Engl J Med **2002**; 346:1300–8.

21. Breman JG. Monkeypox: an emerging infection for humans? In: Scheld WM, Craig WA, Hughes JM, Emerging infections. 4th ed. Washington, DC: American Society for Microbiology, **2000**:45–7.

22. Richet P. L’histoire et l’oeuvre de l’OCCGE in Afrique occidentale francophone. Trans R Soc Trop Med Hyg **1965**; 59: 234–54.

23. Waddy BB. Rural health services in the tropics and the training of medical auxiliaries for them. Trans R Soc Trop Med Hyg **1963**; 57: 384–91.

24. Ogden HG. CDC and the smallpox crusade. Washington, DC: US Department of Health and Human Services, **1987**.

25. Foege WH, Millar JD, Lane JM. Selective epidemiologic control in smallpox eradication. Am J Epidemiol **1971**; 94:311–5.

26. Foege WH. House on fire: the fight to eradicate smallpox. California/Milbank Books Berkeley, CA: University of California Press; **2011**.

27. Brilliant L. Sometimes Brilliant: the impossible adventure of a spiritual seeker and visionary physician who helped conquer the worst disease in history. Reprint ed. HarperOne, **2017**.

28. Hughes K, Foster SO, Tarantola D, Mehta H, Tulloch JL, Joarder AK. Smallpox surveillance in Bangladesh: II - Smallpox facial scar survey assessment of surveillance effectiveness. Int J Epidemiol **1980**; 9:335–40.

29. Breman JG, Arita I. The confirmation and maintenance of smallpox eradication. N Engl J Med **1980**; 303:1263–73.

30. Breman JG, Arita I, Fenner F. Preventing the return of smallpox. N Engl J Med **2003**; 348:463–6.

31. Petersen BW, Damon IK, Pertowski CA, et al. Clinical guidance for smallpox vaccine use in a postevent vaccination program. MMWR Recomm Rep **2015**; 64:1–26.

32. McCurdy LH, Larkin BD, Martin JE, Graham BS. Modified vaccinia Ankara: potential as an alternative smallpox vaccine. Clin Infect Dis **2004**; 38:1749–53.

33. Lane JM. The current and future landscape of smallpox vaccines. Global Biosecurity **2019**; 1:106–12.

34. SIGA Technologies. US Food and Drug Administration approves SIGA Technologies’ TPOXX® (tecovirimat) for the treatment of smallpox. 13 July **2018**. https://investor.siga.com/news-releases/news-release-details/us-food-and-drugadministration-approves-siga-technologies. Accessed 20 October 2020.

35. Noyce RS, Lederman S, Evans DH. Construction of an infectious horsepox virus vaccine from chemically synthesized DNA fragments. PLoS One **2018**; 13:e0188453.

36. Rimoin AW, Mulembakani PM, Johnston SC, et al. Major increase in human monkeypox incidence 30 years after smallpox vaccination campaigns cease in the Democratic Republic of Congo. Proc Natl Acad Sci U S A **2010**; 107:16262–7.

37. Yinka-Ogunleye A, Aruna O, Dalhat M, et al; CDC Monkeypox Outbreak Team. Outbreak of human monkeypox in Nigeria in 2017-18: a clinical and epidemiological report. Lancet Infect Dis **2019**; 19:872–9.
